# Precision of automatically generated generic regions of interest for direct assessment of perfusion-MRI

**DOI:** 10.3389/fphys.2026.1829907

**Published:** 2026-05-21

**Authors:** Stephan Dehen, Christian Nasel

**Affiliations:** 1 Department of Diagnostic and Interventional Radiology, University Hospital Tulln, Karl Landsteiner University, Tulln, Austria; 2 Department of Medical Imaging and Image-guided Therapy, Medical University of Vienna, Vienna, Austria

**Keywords:** automatic segmentation, cerebral perfusion imaging, magnetic resonance imaging, SPM12, standardized time to peak

## Abstract

**Background and purpose:**

Quantitative perfusion measurement using magnetic resonance imaging (P-MRI) is an important method to detect cerebral pathologies. An accurate measurement of quantitative perfusion parameters in correctly placed regions of interest (ROIs) is mandatory to distinguish between regular and pathological tissue perfusion. Especially in large cohorts, the current gold-standard of manually drawn ROIs is time-consuming, and comparisons of different examinations require an additional transformation of perfusion parameter maps from individual native to the so-called standard space, which additionally confounds the quantitative measurements due to necessary spatial interpolations, which potentially introduces substantial error into the measurements. Therefore, we propose an automatic reverse transformation (arT) method that projects generic ROIs from standard to native space, which enables direct assessment of the original quantitative data in native space.

**Materials and methods:**

P-MRI data from 36 subjects without detectable lesions were manually segmented in native space using seven predefined ROIs. The same ROIs were manually drawn in standard space using a high-resolution MNI template, and after applying arT, spatial overlap with manual ROIs was assessed using the Sørensen-Dice index (SDI). Furthermore, the impact from spatial overlap error on quantitative perfusion parameters (standardized Time-to-Peak [stdTTP], cerebral blood volume [CBV]) was evaluated.

**Results:**

Depending on ROI size, the SDI ranged from 0.766 (right basal ganglia) to 0.948 (right cerebral hemisphere). For evaluation of stdTTP, the bias was -0.002 s, with limits of agreement from -0.160 s to 0.138 s and Pearson correlation coefficient between 0.946 and 0.999. Similar correlations were observed for CBV.

**Conclusions:**

Given the acceptably small relative variability (automatic vs manual) and low error of absolute measurements, arT could become a promising tool for the automatic assessment of quantitative cerebral P-MRI parameters.

## 1 Introduction

Assessment of cerebral perfusion using magnetic resonance imaging (MRI) proved useful in diagnostics of various cerebral pathologies, above all in the detection of acute ischemic stroke. Among other MRI-based perfusion methods, dynamic susceptibility contrast (DSC)-perfusion-MRI (P-MRI), which employs Gd-based contrast agents as tracers, seems most favorable, as it offers the highest temporal resolution at the shortest examination times with maximal contrasts. DSC-MRI provides several perfusion parameters, such as standardized time to peak (stdTTP) or cerebral blood volume (CBV), which allow quantification of perfusion alterations in selected regions of interest (ROIs). Many of these parameters use predefined thresholds, which allow distinction between regular and critical perfusion. For instance, with the stdTTP-parameter values > 7 s are considered as critical, where a reliable and exact separation of regular from critical perfusion is mandatory (Nasel et al., 2000; Nasel et al., 2004).

The distinction between physiological and pathological conditions often requires placement and assessment of ROIs. This can be done either manually or (semi-) automatically using various methods, e.g., intensity-based, surface-based, application of machine/deep learning, or atlas-based (Despotovic et al., 2015; Singh and Singh, 2021). However, manual segmentation, currently considered as the gold standard, is prone to bias from inter- or intra-observer differences and rapidly becomes impractical when large datasets need to be segmented (Collier et al., 2003; Dijkstra et al., 2020). Automatic techniques, employing machine learning and deep learning models, may also be limited in segmenting neuroanatomic structures, such as, e.g., convolutional neural networks (CNNs), which may lose meaningful information when working with 2D slices, and using 3D volumetric data could rapidly require excessive computational power (Chen et al., 2018; Huo et al., 2019; Feng et al., 2020). Though newer methods, such as SLANT or AssemblyNet, try to overcome this problem by utilizing multiple sub-specialized CNNs, these models tend to struggle when confronted with pathologies, since handling a greater anatomical variability also demands considerably higher amounts of additional training data (Glasser et al., 2016; Huo et al., 2019; Coupe et al., 2020; Andrews and Di Ieva, 2025). Therefore, using more robust (multi-) atlas-based methods, which rely on predefined intensity images, i.e., templates, in standard space, provided together with aligned segmented (labeled) topological or probabilistic images, which can also handle large datasets, seems currently most practical (Aljabar et al., 2009; Cabezas et al., 2011). Moreover, to a certain degree, these methods additionally allow some sample-specific adaptions, because suitable templates can be chosen from a huge variety for different populations, anatomical regions, or imaging modalities (Iglesias and Sabuncu, 2015). However, this method requires alignment between the original MR images and a template, which is achieved by transforming the originally measured data from native space to the template’s standard space using a pipeline usually consisting of co-registration, image segmentation and spatial normalization, where the final analysis is then performed in the generic space (Ashburner and Friston, 2005). Though rather robust, this approach could also produce significant errors, if, for example, spatial normalization slightly overestimates the intracerebral volume (Giff et al., 2023), which increases the partial volume error (Aribisala et al., 2011; Hutchison et al., 2014). However, the major drawback of this method is the need to resample and interpolate the original data during the transformation from native to standard space, which introduces considerable uncertainties and artifacts, but, above all, this alters the absolute values of the original measurements (Grootoonk et al., 2000). Most of improvements suggested for these transformations aimed to solve problems of spatial alignment, but widely neglected the inevitable bias introduced to the manipulation of the absolute parameter values of the original measurements. In contrast to this, we propose direct assessment of absolute parameter values in original native space using generic ROIs after automatic reverse transformation (arT) from standard to native space. The arT-method should, therefore, enable an unbiased direct evaluation of functional parameter maps in native space, as it does not directly affect the measured absolute values. Although this overcomes any confounding of originally measured absolute values by interpolations taking place in the parameter map during the usual spatial transformation procedure to standard space, arT could still be prone to bias introduced by inconsistencies in the spatial transformation of the generic ROIs from standard to native space. Therefore, we systematically investigated the magnitude of the error introduced by the arT-approach in the assessment of quantitative perfusion parameter maps generated using DSC-MRI. To the best of our knowledge, limitations and bias introduced by arT in the assessment of P-MRI-derived functional parameter maps using atlas-based generic ROIs was not been investigated so far. Therefore, we compared spatial and magnitude properties of generic ROIs, previously defined in standard space, after arT with corresponding manually drawn ROIs in the same subject in native space.

## 2 Materials and methods

### 2.1 Patients

Multi-parametric MRI examinations of 36 consecutive patients (16 female and 20 male; age: 49 ± 14.9 years) between June 1^st^ 2022 and May 31^st^ 2024 were extracted from our institutional database, which were performed to exclude acute cerebrovascular disease, but turned out as “non-ischemic/lesional”. Prior to the analysis, all imaging data were anonymized and sent to a workstation for further assessment.

This study was performed according to current standards defined by the World Medical Association (2025). Approval was granted by the Ethics Committee of Karl Landsteiner University of Health Sciences (number 1054/2024). Informed consent was waived due to the retrospective character.

### 2.2 Magnetic resonance imaging

**Figure 1:**
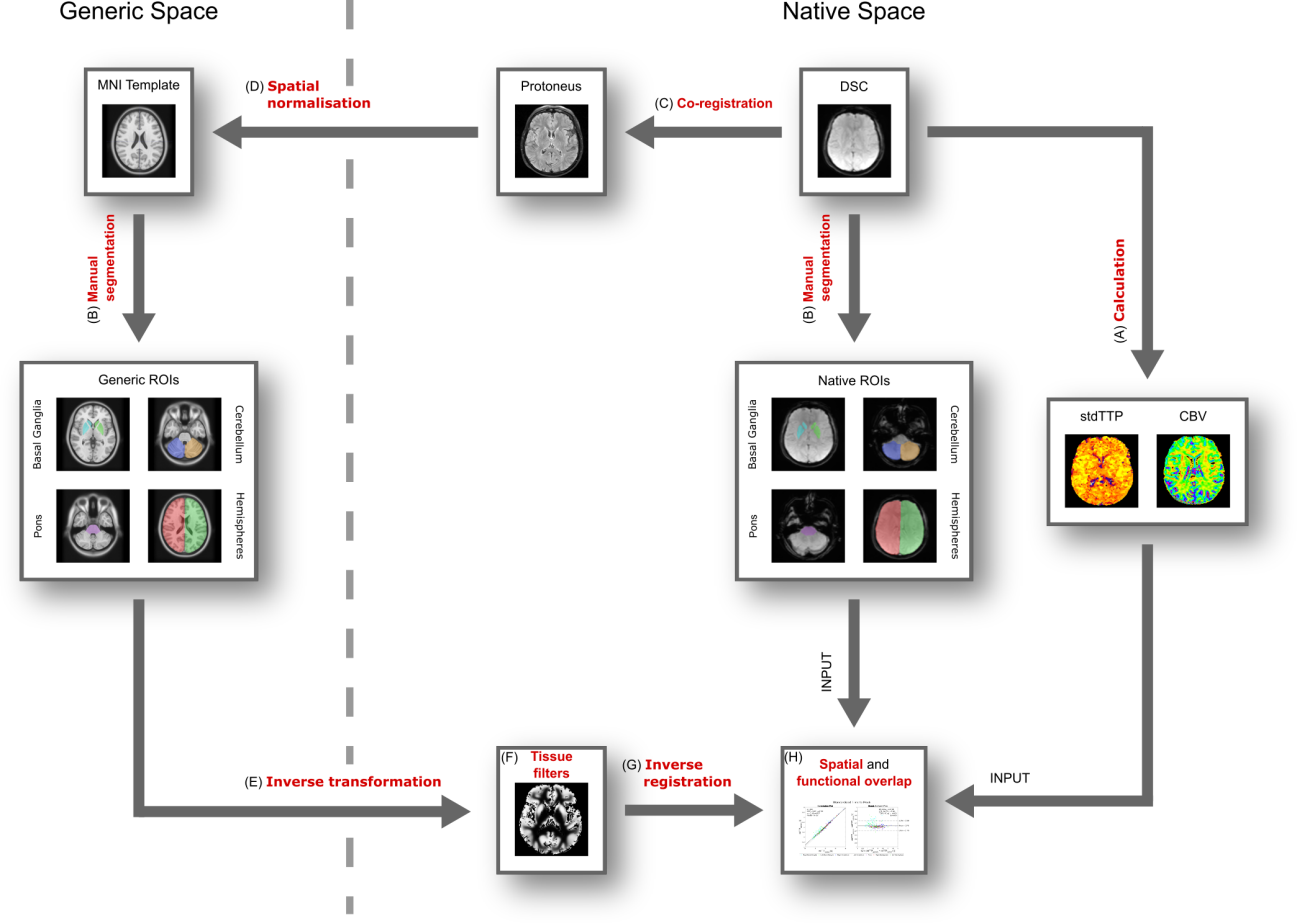
Flowchart of the study. **(A)** Calculation of the stdTTP- and CBV-maps based on the perfusion MRI (DSC). **(B)** Manual segmentation of the ROIs in the generic and native templates. **(C)** Co-registration of the DSC-sequence to the high resolution Protoneus sequence. **(D)** Spatial normalization of the Protoneus sequence to the generic template. **(E)** Inverse transformation of the generic ROIs to the native spaces. **(F)** Filtering of the transformed generic ROIs based on tissue distribution. **(G)** Inverse registration of the filtered and transformed generic ROIs to the DSC sequence. **(H)** Calculation of spatial and functional overlap.

All examinations were performed on a clinical 1.5 T MR scanner (Avanto FIT SQ, Siemens Medical Systems, Erlangen, Germany). DSC-MRI was performed using a dynamic contrast-enhanced T2*-weighted single shot gradient echo multi-band echo planar imaging sequence (TE = 17 ms, TR = 689 ms, flip angle = 35°). This way, 81 stacks with 20 slices and a voxel size of 1.154 x 1.154 x 6 mm (gap = 1 mm) at a scan time of 60 seconds were generated. If necessary, to account for patient movement during acquisition, the stacks were additionally linearly realigned prior to further assessment. After this, perfusion parameter- and event- maps were generated using a scientific standard software (jPerfusionModule, C. Našel, V 3.2), which provided the raw data information of the time-distribution-curve (TDC) of the measurement and quantitative standardized time to peak- (stdTTP), cerebral blood volume- (CBV), and mean-TDC- maps (**Figure 1A**). In short, the TDC-maps reflect the course of the whole perfusion event and give also an anatomical so-called mean image of the whole event, while stdTTP is calculated by voxel-wise referring absolute TTP-values in each measured slice of the T2*-weighted sequence to a previously computed slice-specific offset. Corresponding CBV-maps were calculated voxel-wise by integrating and normalizing the time-contrast-curves in the time interval of the TDC-perfusion event derived from the DSC-measurement. More details of all calculations can be found elsewhere (Nasel et al., 2014; Nasel et al., 2017; Nasel et al., 2019). In addition to DSC-MRI, the routine imaging protocol also included a high-resolution enhanced PD/T2w-IR-sequence (Protoneus-sequence) with a voxel size of 0.5 x 0.5 x 6.0 mm (Nasel, 2005) and a dual-b (b = 0 & 1000 s/mm^2^) diffusion weighted spin echo-EPI sequence with a voxel size of 1.25 x 1.25 x 6.0 mm, both performed spatially matched to the DSC-measurement.

### 2.3 Image processing

**Figure 2:**
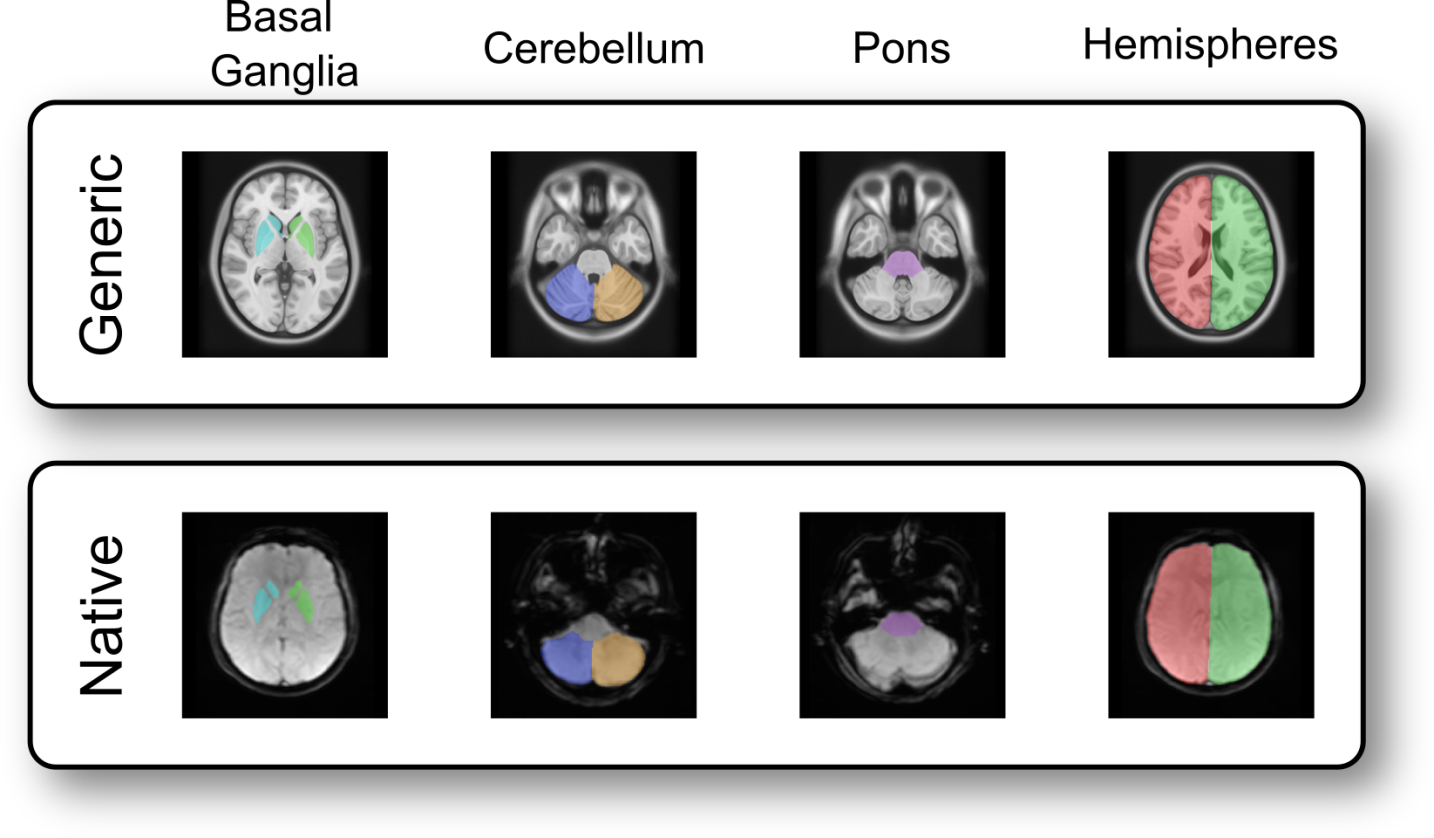
Seven representative ROIs which were defined and drawn in generic standard space and individual native space. The upper row displays the ROIs in the generic template, while the lower row shows the same ROIs in one of the assessed patients.

Seven exemplary ROIs of various sizes covering the right/left basal ganglia, the pons, the right/left cerebellar hemispheres, and the right/left cerebral hemispheres were defined (**Figure 2**) and manually drawn in consent by two experts (35 years and 3 years of experience in Neuroradiology) in generic standard- and individual native-space images, respectively (**Figure 1B**) using freely available standard software (ITK-Snap, Version 4.2.0) (Yushkevich et al., 2006). This set of ROIs was chosen to gain a representative mix of small and large sized as well as supra- and infratentorial structures in order to assess effects from size and location of the ROIs on arT. The process of optimally covering the selected structures with ROIs ended, however, when both readers agreed to have reached their best matching case-specific result. Manual segmentation in the individual native-space was, thereby, defined as “gold standard”. As generic standard-space, the open source ICBM152 2009a nonlinear asymmetric MNI template with a spatial resolution of 1 x 1 x 1 mm was chosen (NeuroImaging & Surgical Technologies Lab, McGill University, Canada) (Fonov et al., 2009; Fonov et al., 2011).

After delineating the individual native space ROIs and generating the generic ROI-template, the mean-TDC images from DSC-MRI were linearly co-registered to the Protoneus sequence, where the space-defining Protoneus sequence served afterwards to calculate the spatial non-linear transformation matrices to and from (= inverse transformation matrix) MNI-standard space in each patient using the SPM12 segmentation tool (**Figures 1C, D**). Beside the spatial transformation matrices, this step also provided tissue probability maps of gray matter, white matter, and cerebrospinal fluid (CSF), which were used later to filter the reversely transformed generic ROIs to reduce spatial outliers. Using these individual inverse transformation matrices, all generic standard-space ROIs were then inversely transformed backwards into original native space defined by the individual Protoneus-sequences (**Figure 1E**). Filtering of the transformed generic ROIs (**Figure 1F**) was performed by adding the probabilities for gray and white matter in each voxel, where only voxels above a 10% tissue probability threshold were kept (Rorden et al., 2012). Only in the assessment of the right and left cerebral hemispheres, additionally, the likelihood of the cerebrospinal fluid spaces was considered, and voxels with a combined probability above 10% were kept. Lastly, the generic ROIs were reversely transformed back into native space in all patients (**Figure 1G**). The resulting corresponding pairs of transformed generic and manually drawn native ROIs were then used for further comparisons (**Figure 1H**). The whole image processing procedure was implemented using the software package SPM12 (Functional Imaging Laboratory, UCL Queen Square Institute of Neurology, London, UK) based on MATLAB (R2024b, MathWorks, Natick, USA).

### 2.4 ROI – image assessment and statistical analysis

The spatial deviation of generic and native ROIs was assessed by calculation of the volume error (VE) defined as:

(1) $$\begin{array}{c} VE=\frac{V_{native}-\;V_{generic}}{0.5\left(V_{native}+V_{generic}\right)}, \end{array}$$

where V_native_ and V_generic_ denote the volume of the ROIs (Yaakub et al., 2020). For all ROIs, additionally, the surface-to-volume ratio (SVR) is given. The quality of the spatial match between individual corresponding generic and native ROIs was evaluated on a voxel-by-voxel basis using the Sørensen-Dice index (SDI) (Dice, 1945) as:

(2) $$\begin{array}{c} SDI=\frac{2\left|{\text{V}}_{\text{native}}\cap {\text{ V}}_{\text{generic}}\right|}{\left|{\text{V}}_{\text{native}}\cup {\text{V}}_{\text{generic}}\right|}, \end{array}$$

where V_native_ and V_generic_ have the same meaning as in Equation 1.

Furthermore, the influence of the initial ROI volume and SVR on SDI was explored by linear regression analysis, including Pearson’s Correlation Coefficient (PCC) and Spearman’s Rank Correlation Coefficient (SRCC).

On the subject-level, the dependence of absolute stdTTP- and CBV-values on SDI was assessed by calculating and comparing the means of both parameters in native and generic ROIs, respectively. At the group-level, the strength of the correlation between generic and native values was explored using the PCC and linear regression analysis, including the calculation of the normalized root mean squared error (NRMSE), as shown in equation 3, where *n* denotes the number of included ROIs. Additionally to this, Lin’s concordance correlation coefficient (CCC) was calculated (Lin, 1989).

(3) $$\begin{array}{c} NRMSE=\frac{\sqrt{\frac{1}{n}\sum_{i=1}^{n}{\left(stdTTP_{fit}\left(i\right)\;-\;stdTTP_{nat}\left(i\right)\right)}^{2}}}{\frac{1}{n}\sum_{i=1}^{n}stdTTP_{nat}\left(i\right)} \end{array}$$

Because correlation and linear regression analysis alone potentially fail to detect significant deviations in just a certain range of values when comparing two methods (Grouven et al., 2007; Ranganathan et al., 2017), the deviations of the perfusion parameters were also investigated by Bland-Altmann plots (Bland and Altman, 1986) with calculation of the 95% confidence intervals of the bias (CI_95%_) (Giavarina, 2015). For testing non-inferiority of non-normal-distributed data and using the Wilcoxon signed-rank test (in combination with the error margin Δ), we estimated a sample size of 35 with α = 2.5% and power (1-β) = 80% (G*Power calculator, version 3.1.9.7) (Faul et al., 2009). By subdividing each map into 7 ROIs, thereby risking, in parts, non-independency of the data, we may underestimate uncertainty, but also enable a thorough assessment of the most relevant confounder, i.e., the ROI volume. In this analysis, the limits of agreement (LOA) were set at the 2.5^th^ and the 97.5^th^ percentiles (Gerke, 2020). Descriptive statistics are given as median and median absolute deviation (MAD). All statistical assessments were performed using the software package MATLAB (R2024b, MathWorks, Natick, USA), including the *Statistics and Machine Learning Toolbox* (Version 24.2).

## 3 Results

### 3.1 Spatial overlap

**Figure 3:**
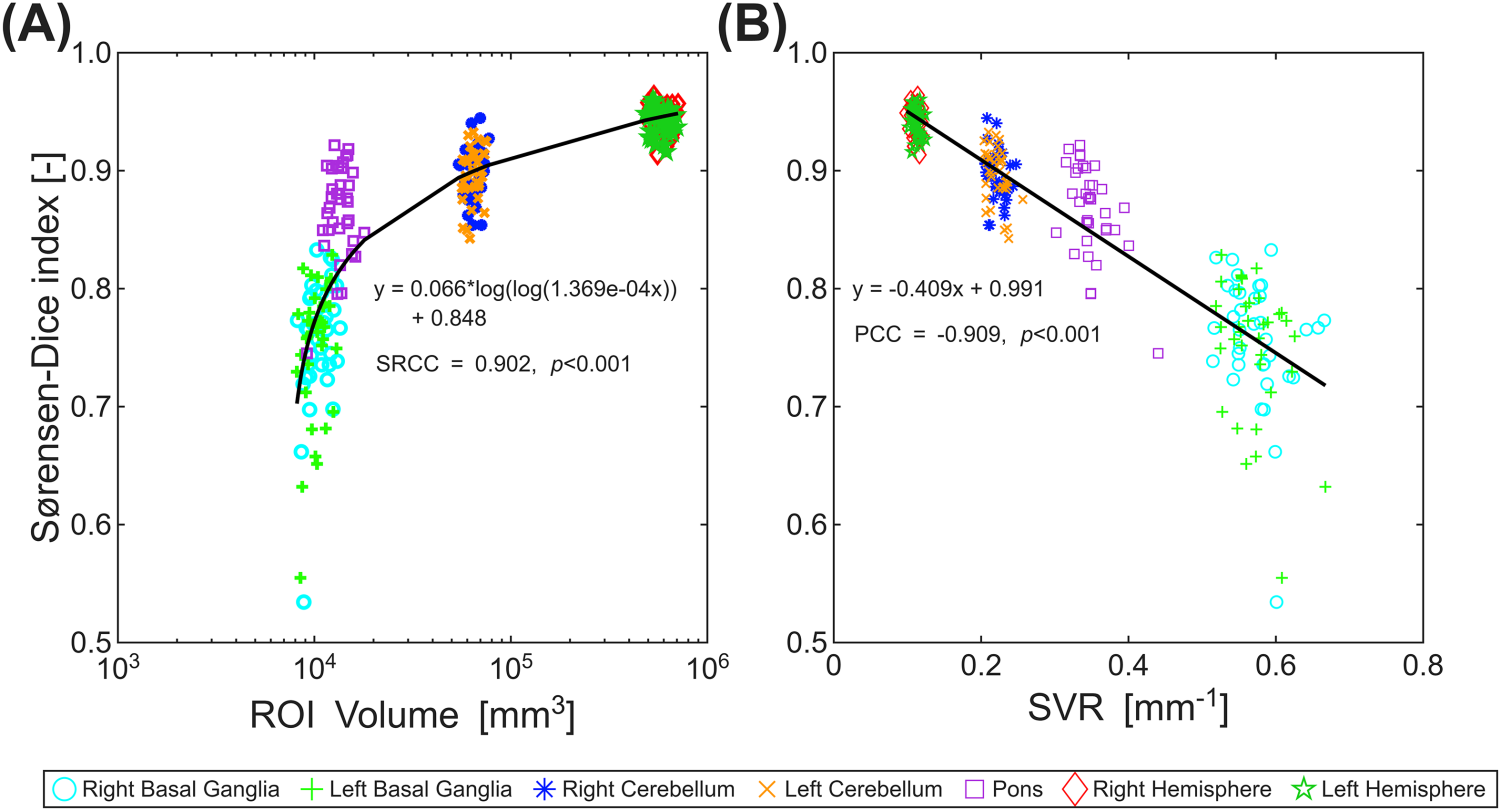
Scatterplots of the Sørensen-Dice index (SDI). **(A)** SDI in relation to the ROI volumes and corresponding correlation function (black line). **(B)** SDI in relation to the surface-to-volume ratio (SVR) of the ROIs and corresponding linear regression function (black line).

Comparing transformed generic and native ROIs, we found higher SDIs of up to 0.948 (right cerebral hemisphere) correlated with larger ROI volumes (**Figure 3**). Assessment of VE indicated a relative oversizing of small volumes by transforming generic ROIs, while manually drawn native ROIs tended to overestimate larger volumes (VE: -4.42% [min: right basal ganglia], +7.92% [max: left cerebellum]). The volumes assessed by the various ROIs ranged from 10.3 10^3^ [min: basal ganglia] to 593.9 10^3^ mm^3^ [max: hemispheres] with an SVR ranging from 0.57 [max: left basal ganglia] to 0.11 [min: hemispheres], where SVR was found to be indirectly proportional to the volume. A summary of the various spatial and volumetric measurements is given in **Table 1**. In spatial assessment, we detected one outlier concerning the generic ROIs (case 34). Closer inspection indicated an artifact in the tissue probability maps induced by CSF-flow during spatial normalization of the Protoneus sequence to the MNI space. This was corrected in this particular case by using the b 0-images from the geometrically matched DWI-sequence. All over, the values of the SDIs showed a non-linear (double logarithmic) relationship to the ROI volume with an excellent SRCC of 0.902 (*p* < 0.001), indicating a monotonous correlation (**Figure 3A**). In comparison to the SVR, SDI showed a strong inverse linear correlation with a PCC of -0.909 (*p* < 0.001).

**Table 1:** Table containing average ROI volumes, surface-to-volume ratios, volume errors, and SDIs. All values are written in the form Median ± MAD.

ROI names	V_mean_^1^ [10^3^ mm^3^]	SVR^1^ [1/mm]	VE^1^ [%]	SDI^1^ [-]
Right Basal Ganglia	10.6 ± 1.2	0.57 ± 0.03	-4.42 ± 5.90	0.766 ± 0.030
Left Basal Ganglia	10.3 ± 1.0	0.57 ± 0.02	-4.31 ± 6.94	0.768 ± 0.024
Right Cerebellum	65.3 ± 4.6	0.22 ± 0.01	5.83 ± 3.88	0.899 ± 0.013
Left Cerebellum	64.3 ± 4.1	0.22 ± 0.01	7.92 ± 4.43	0.899 ± 0.014
Pons	13.5 ± 1.2	0.35 ± 0.01	-4.22 ± 5.68	0.875 ± 0.026
Right Hemisphere	593.9 ± 42.3	0.11 ± 0.00	3.43 ± 3.68	0.948 ± 0.007
Left Hemisphere	587.3 ± 47.2	0.11 ± 0.00	2.49 ± 3.40	0.944 ± 0.007

^1^ Median ± MAD.

### 3.2 Functional parameters

**Figure 4:**
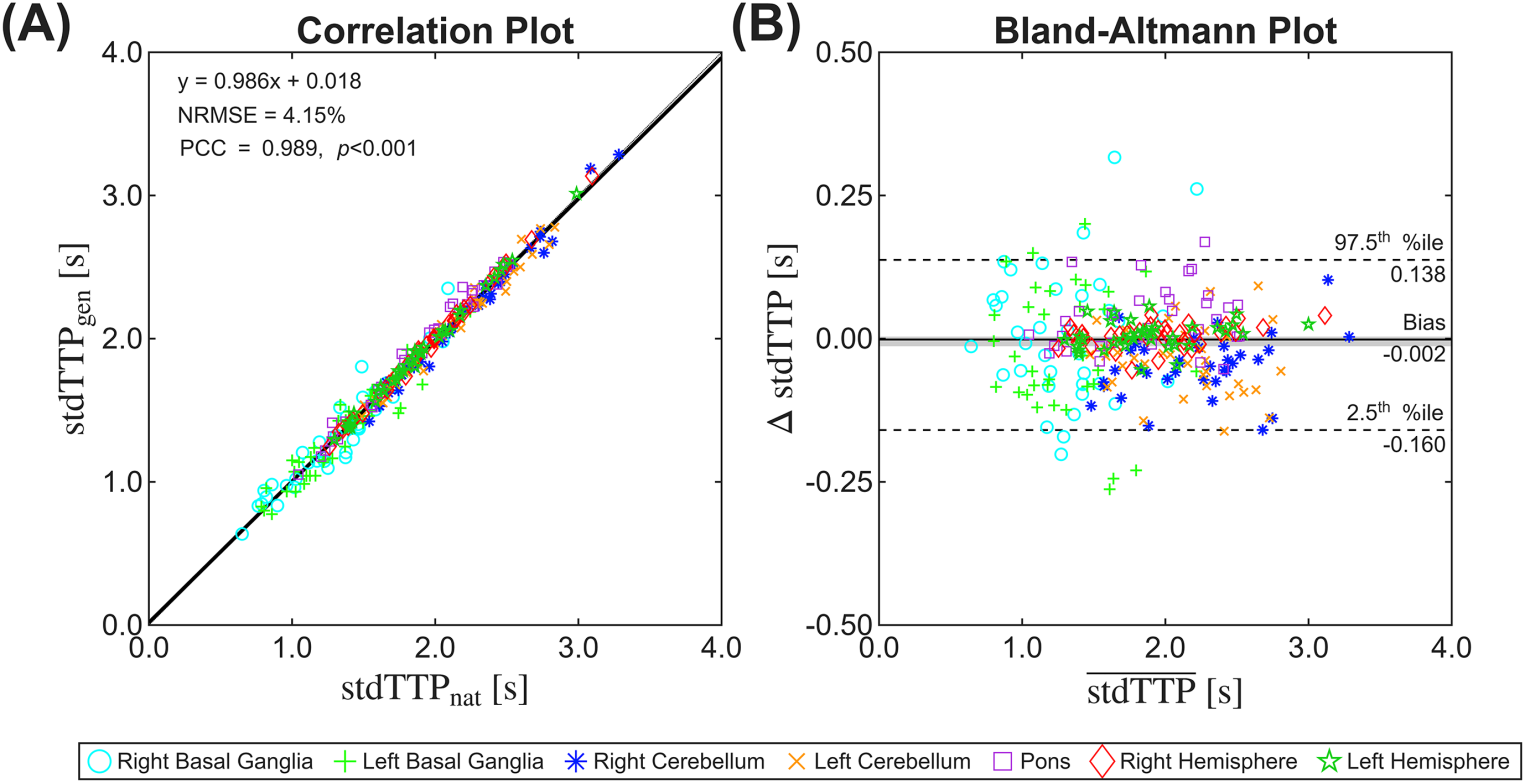
Correlation **(A)** and Bland-Altmann **(B)** plots of stdTTP in all transformed generic and native ROIs (n=252). We found an excellent, strong linear correlation and acceptably low NRMSE. The dashed lines in the Bland-Altmann plot indicate the limits of agreement (LOA), while the solid line and shaded area indicate the bias and its CI95%. Of note, homoscedasticity for all measurements can be derived from the plot.

**Figure 5:**
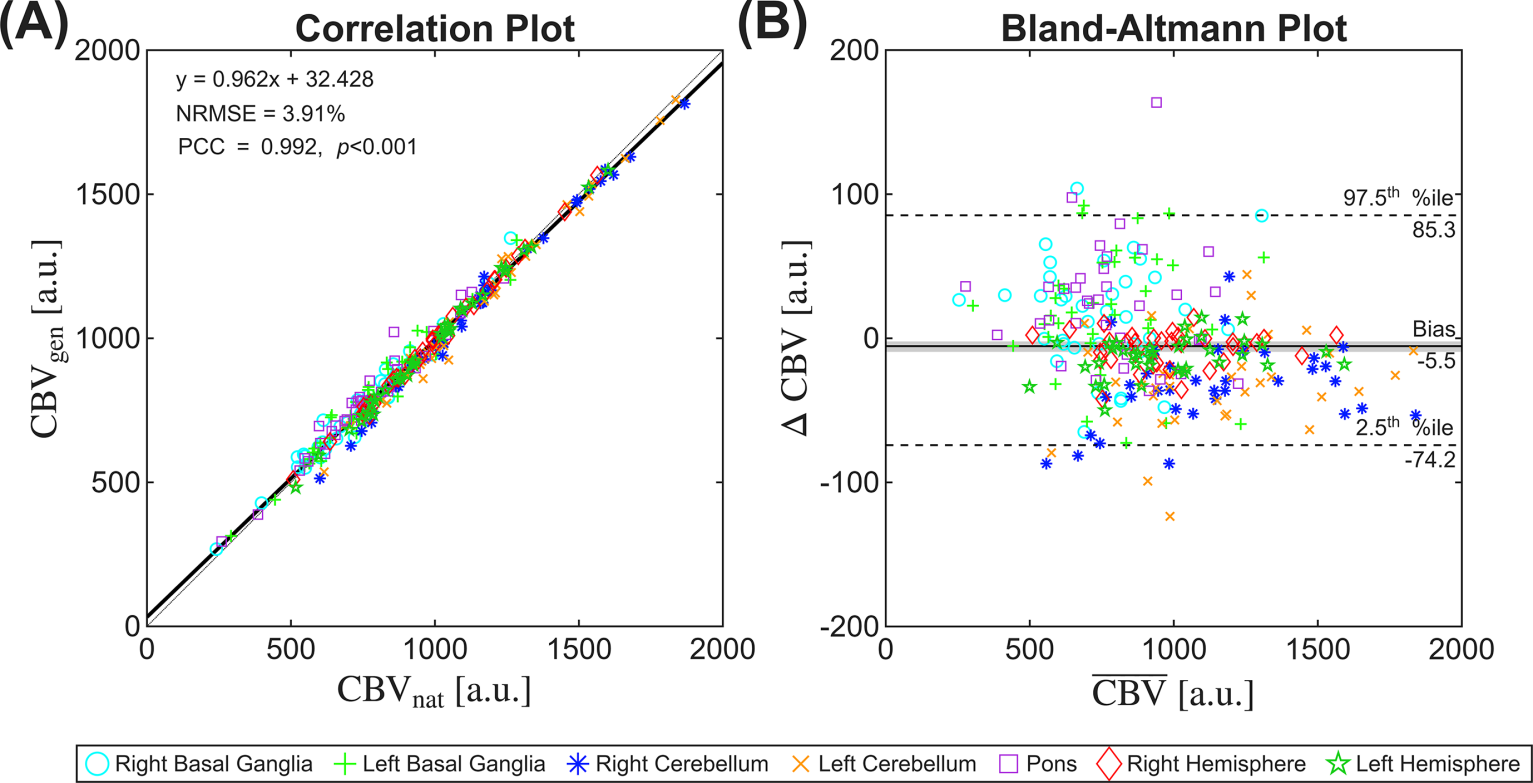
Correlation **(A)** and Bland-Altmann **(B)** plots of CBV (a.u.: arbitrary units) in all transformed generic and native ROIs (n=252). Compared to stdTTP, the CBV measurements showed an excellent strong correlation between transformed generic and native ROIs with only a small NRMSE. The dashed lines in the Bland-Altmann plot indicate the limits of agreement (LOA), while the solid line and shaded area indicate the bias and its CI95%. As is visible, the plot proved homoscedasticity for all measurements.

Comparing the functional parameter values between native and transformed generic ROIs showed all values for stdTTP (**Table 2**) and CBV (**Table 3**) within the postulated physiological range in each ROI. Expectedly, the median values of stdTTP were in the postulated regular range, with values between 1.292 s (basal ganglia) and 2.273 s (cerebellum). Linear regression analysis of stdTTP-measurements revealed a PCC of *r* = 0.989 (*p* < 0.001), and the NRMSE of the linear fit was found at 4.15% (**Figure 4A**). The concordance correlation coefficient demonstrated an excellent agreement between generic and native ROIs (0.944 - 0.999 [range]). The Bland-Altmann plot proved homoscedasticity for all stdTTP-measurements (**Figure 4B**). The LOA ranged from -0.160 s to 0.138 s for all ROIs. The total bias for stdTTP-measurements was -0.002 s (-0.046 s - 0.025 s [range]) with a CI_95%_ of the bias of [-0.013, 0.004], which includes the line of equality. Therefore, no significant difference could be shown between the two methods. For the cerebral blood volume (CBV), linear regression analysis exhibited a PCC of r = 0.992 (*p* < 0.001), and the NRMSE of the linear fit was found to be 3.91% (**Figure 5A**). Comparable to stdTTP, the CCC indicated an excellent agreement between transformed generic and native ROIs (0.975 - 0.998 [range]), while the Bland-Altmann plot also proved homoscedasticity for all CBV measurements (**Figure 5B**). The LOA ranged from -74.2 to 85.3 a.u. with a bias of -5.5 a.u. and a CI_95%_ which does not include the line of equality. The LOA ranged from -74.2 a.u. to 85.3 a.u. combined. The total bias of CBV measurements was -5.5 a.u. (-31.1 - 25.1 a.u. [range]). The CI_95%_ of the bias for CBV [-9.4 - -2.0 a.u.] failed to include the line of equality.

**Table 2:** Results of the functional overlap between generic and native ROIs in terms of stdTTP.

ROI names	stdTTP_nat_^1^ [s]	stdTTP_gen_^1^ [s]	Bias^1^ [s]	PCC^2^ [-]	CCC^2^ [-]
Right Basal Ganglia	1.323 ± 0.229	1.242 ± 0.262	0.002 ± 0.075	0.951	0.950
Left Basal Ganglia	1.292 ± 0.245	1.241 ± 0.228	-0.030 ± 0.077	0.946	0.944
Right Cerebellum	2.273 ± 0.344	2.229 ± 0.379	-0.046 ± 0.028	0.993	0.987
Left Cerebellum	2.179 ± 0.317	2.138 ± 0.318	-0.036 ± 0.043	0.988	0.984
Pons	1.935 ± 0.329	1.966 ± 0.362	0.025 ± 0.035	0.993	0.990
Right Hemisphere	1.956 ± 0.241	1.945 ± 0.246	0.009 ± 0.010	0.999	0.999
Left Hemisphere	1.848 ± 0.238	1.831 ± 0.224	0.005 ± 0.016	0.998	0.998
Total	1.797 ± 0.394	1.793 ± 0.393	-0.002 ± 0.040	0.989	0.989

^1^ Median ± MAD.

^2^ p<0.001.

**Table 3:** Results of the functional overlap between generic and native ROIs in terms of CBV.

ROI names	CBV_nat_^1^ [a.u.]	CBV_gen_^1^ [a.u.]	Bias^1^ [a.u.]	PCC^2^ [-]	CCC^2^ [-]
Right Basal Ganglia	736.6 ± 125.3	728.1 ± 127.2	19.3 ± 23.2	0.983	0.980
Left Basal Ganglia	742.6 ± 142.2	756.0 ± 152.0	20.3 ± 24.7	0.980	0.976
Right Cerebellum	1125.3 ± 201.3	1091.7 ± 208.7	-31.1 ± 17.8	0.996	0.991
Left Cerebellum	1111.2 ± 159.4	1092.8 ± 187.5	-30.6 ± 20.6	0.994	0.989
Pons	746.3 ± 118.6	782.7 ± 115.5	25.1 ± 24.0	0.982	0.975
Right Hemisphere	953.4 ± 159.3	943.9 ± 166.3	-3.3 ± 5.6	0.999	0.998
Left Hemisphere	929.0 ± 146.7	917.7 ± 159.0	-11.2 ± 7.6	0.997	0.997
Total	904.4 ± 181.3	907.7 ± 180.8	-5.5 ± 21.3	0.992	0.991

^1^ Median ± MAD.

^2^ p<0.00.1.

## 4 Discussion

Group-based analysis of regional changes of quantitative functional MRI measurements, like analysis of regional perfusion using DSC-P-MRI, requires either individually drawing many ROIs in certain regions in each patient in native space or transforming all MRI data from native to standard space and to perform the intended analysis after considerable manipulation of the originally measured data. Therefore, assessment of large group data in standard space is tempting, because the finally assessed data in standard space was considerably manipulated by resampling and interpolation during the transformation process. This bears a certain risk that, depending on the respective transformation procedure, subtle but clinically meaningful aspects of the initial real-world measurements could get lost. For instance, the simple and robust nearest-neighbor approach may widely preserve original data values, but it is also prone to block artifacts and provides less precise spatial fitting of the data to the aimed standard space compared to more advanced techniques like tri-linear or b-spline fitting (Meijering et al., 2001). Also, the possible introduction of partial volume errors during spatial normalization could significantly affect the quality of a subtle cerebral perfusion analysis, especially when analyzing absolute data from time-based parameters like the Time-to-Peak parameter (McGehee et al., 2012; Chappell et al., 2021). As shown in this study, using the proposed arT-method, most of these problems could effectively be avoided, since the final assessment of all quantitative functional data is performed in native space, thereby leaving the originally measured perfusion values unchanged.

Analyzing and comparing a total of 252 generic spatially transformed standard- and individually drawn native-space ROIs in seven different regions of the brain in 36 subjects without detectable brain lesions, we found evidence that especially the assessment of time-based perfusion parameters using the arT-method is equivalent to the ‘gold standard’ of manually drawing ROIs in these brain structures in each individual patient. However, our results also urge caution when assessing primarily signal-based parameters, like the CBV-parameter, where full equality between the generic and the manual ‘gold-standard’-method could not be shown. Nevertheless, as the overall agreement between generic and native measurements was excellent, by using the proposed arT-method, which requires only one generic ROI-template that is transformed into the original native space of each tested subject, an automatic assessment of large numbers of patients without significant loss of precision of the perfusion measurements seems achievable.

### 4.1 Spatial overlap

SDI generally ranged between 0.766 in the basal ganglia and 0.948 in the hemispheres. As a correlation of 0.7 is usually considered the threshold for a good match, our results suggest an excellent match between native- and generic-ROIs (Zijdenbos et al., 1994). The relatively lower spatial overlap found in the basal ganglia could have several reasons. Firstly, it could be shown (**Figure 3**) that the SDI decreases with the volume size of the analyzed ROI, which is also described by Isambert et al. (2008). Hence, with bigger ROIs small overlap-errors at the borderlines will have a negligible impact on the measurements only. Secondly, the manual segmentation of the basal ganglia in the used mean images of DSC-sequences was partially hampered by low contrast, hence mainly the co-registered Protoneus sequences were used. Thirdly, the relatively low resolution in z-direction (6.0 mm) could have introduced a relevant partial volume artefact, especially in smaller structures like the basal ganglia, which were depicted only on 2-3 slices in the native-space images, thereby introducing a rather high degree of uncertainty. Nevertheless, the overall performance of the proposed arT-method with an SDI of 0.766 seems reliable and is in accordance with values reported in literature. Using the same software package as in this study for segmenting the thalamus and hippocampus, SDIs of 0.787 and 0.735 could be achieved (Naess-Schmidt et al., 2016). Also, the use of a different software package for segmentation of the globus pallidus, the putamen, and the caudate nucleus revealed comparable SDI-values (Velasco‐Annis et al., 2017). Comparable to this, although the ROI delineating the pons also consisted only of approximately 3-4 slices, the preferential orientation of this structure along the z-axis in axial MRI slices enabled a much better demarcation against adjacent tissues than in the basal ganglia. Hence, the SDI found for the pons was clearly higher with 0.875, which was similar to other studies, which reported SDIs of 0.85, 0.86, and 0.93 (Isambert et al., 2008; Velasco‐Annis et al., 2017; Sander et al., 2019). The same is true for the cerebellum, showing an SDI of 0.899, where values reported in the literature ranged between 0.82 and 0.94 (Isambert et al., 2008; Park et al., 2014; Weier et al., 2014; Romero et al., 2017). For the segmentation of a whole hemisphere, literature is rather sparse. Comparing automatic segmentation of intracranial brain volumes also using a reversed brain mask (SPM12) with manual segmentation, an SDI of 0.93 was found (Keihaninejad et al., 2010). This was confirmed later using the same approach, where an SDI of 0.96 was reported (Hansen et al., 2015). However, as the available reported values excellently agree with our measurements, the proposed arT-method seems to be a reliable tool for automatic assessment of perfusion-parameter maps.

### 4.2 Functional overlap

Our results indicate a tendency to lower stdTTP-values in generic ROIs than in the native ones. This could result from small errors induced by the still necessary spatial interpolation of the generic data to the new voxel matrix, which could potentially move original generically correct ROI borders from or towards adjacent cerebral major arteries located near the basal ganglia or the pons. Consecutively, partial volume artefacts could, therefore, have falsely labeled parts of adjacent major arteries, which in turn could have significantly reduced a time-dependent perfusion parameter, like stdTTP. Furthermore, we found that the accuracy of the functional overlap for both stdTTP and CBV depends heavily on the spatial overlap. ROIs with a high SDI, e.g., left/right hemispheres, show a higher PCC and CCC, while ROIs with a lower SDI, e.g., left/right basal ganglia, show a lower PCC and CCC. As the SDI correlates mainly with ROI volume (as described above), we consider ROI volume to be the major confounder for functional accuracy. Literature about the influence of ROI segmentation of perfusion parameter maps in general and especially stdTTP and CBV in particular, is extremely rare. However, studies focusing on the effect of automatic segmentation of myocardial P-MRI could serve as a reference (Weng et al., 2010; Xue et al., 2020; Jacobs et al., 2021; Kim et al., 2023; Yalcinkaya et al., 2024). These studies used a DCE-MRI T1-sequence technique, and segmentation was performed either ROI-, template-, or neural network-based. Comparing the relative bias of the available cardiac measurements of the blood flow and perfusion reserve to the relative bias of our results is rational and reveals relative biases ranging from 0.3% (Jacobs et al., 2021) to 3.2% (Yalcinkaya et al., 2024) in terms of myocardial blood flow and from 1.46% (Kim et al., 2023) to 2.8% (Jacobs et al., 2021) for myocardial perfusion reserve, respectively. These results excellently match our measurements. For the stdTTP-parameter, the bias ranges from -0.046 s to 0.025 s, resulting in a total relative bias of -0.08%. For the CBV parameter, the total relative bias was -0.60%, respectively. Since the relative bias in our study is significantly lower than the results reported for alternative methods in the literature, this further indicates that the proposed arT-method potentially generates valid results in the automatic assessment of cerebral perfusion analysis.

### 4.3 Application in non-contrast perfusion MRI

In this study, we used the contrast-agent based DSC-P-MRI-method to generate functional parameter maps to investigate the practicability of arT. This was owed to the fact that especially stdTTP provides reproducible quantitative data of cerebral perfusion dynamics, which facilitated the comparisons of absolute ROI-values. However, arT is not at all specific for DSC-P-MRI, but could also be integrated in existing assessment pipelines using other functional methods, like arterial spin labeling (ASL). This could allow use of arT also in studies where administration of MR-contrast agents is restricted, for instance, due to renal impairment or known drug-specific allergic reactions of the participants (Vella et al., 2025). To this end, simply the native space-defining DSC-raw data images in **Figure 1A** could be replaced by those from the alternative method. We expect an easy integration, particularly of ASL data, since spatial registration and normalization between ASL images and generic space (e.g., MNI), especially when using SPM, was already reported in several studies analyzing healthy subjects or patients with Alzheimer’s or Parkinson’s disease, as well as in patients suffering epilepsy, acute or subacute hemorrhages, or gliomas (Noguchi et al., 2015; Suo et al., 2019; Mutsaerts et al., 2020; Pellerin et al., 2021; Soman et al., 2021; Rahimzadeh et al., 2023). As in protected populations, preferably non-contrast medium based techniques should be applied; the potential compatibility of arT with ASL could also gain importance in studies including children, e.g., for the investigation of pediatric Moyamoya Disease, of attention−deficit/hyperactivity disorders, or in pediatric craniosynostosis (Blauwblomme et al., 2016; de Planque et al., 2021; Gonchigsuren et al., 2022).

### 4.4 Application in the presence of brain lesions

**Figure 6:**
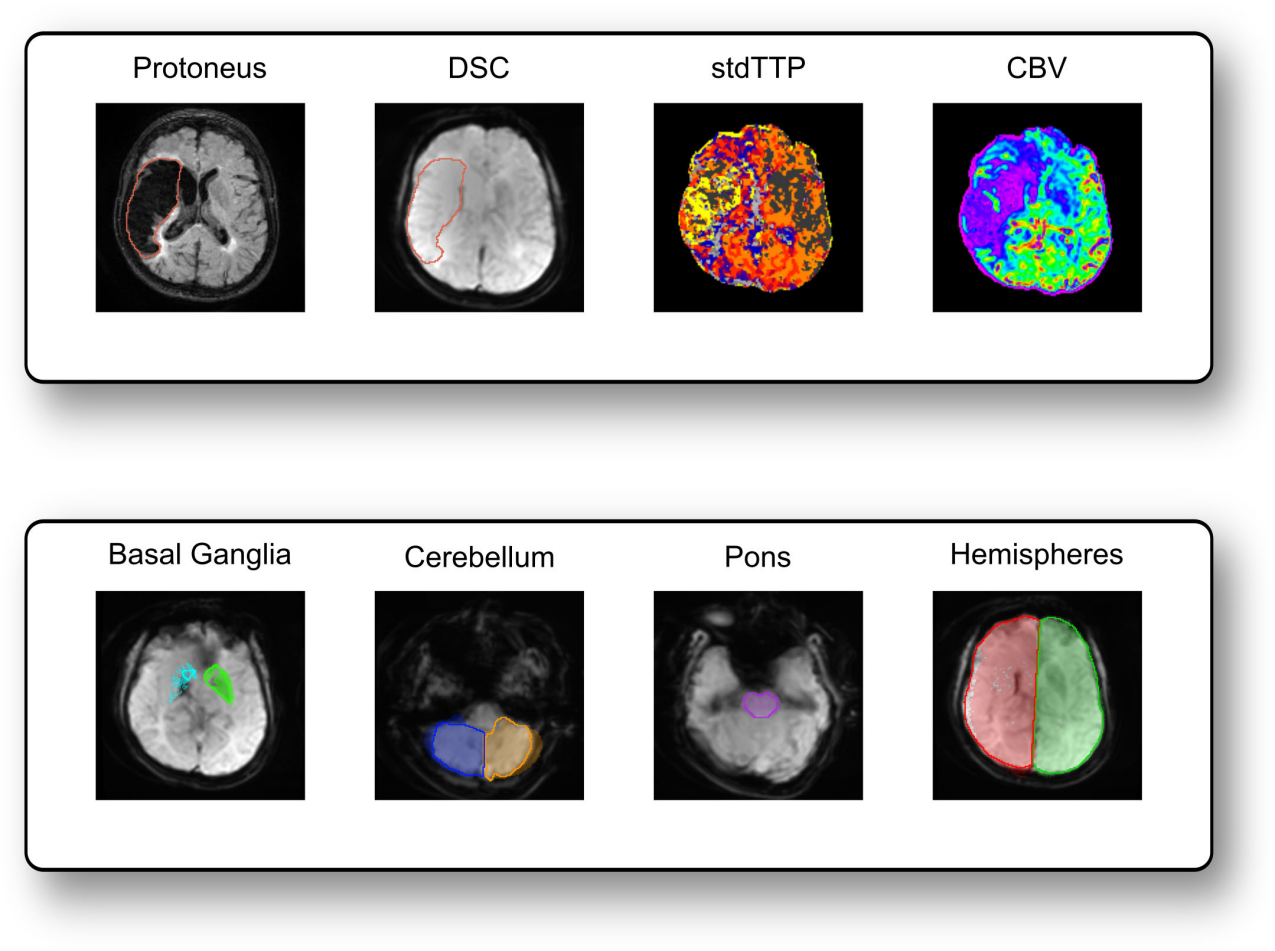
Example of a patient with large chronic stroke (not in the study). The first row depicts the anatomic Protoneus sequence, as well as the DSC mean image with its corresponding stdTTP and CBV maps. The stroke area is delineated in red. The lower row displays the native ROIs (bold circumference) and the corresponding transformed generic ROIs (transparent). The arT-method worked well in this patient even on large lesions and did not require lesion masking.

Although arT is advantageous in studies of large cohorts of patients with diffuse, usually non-lesional, hemodynamic impairments, quite frequently, P-MRI is performed in patients with focal brain lesions (**Figure 6**). As a generic brain atlas cannot directly match an individual brain lesion, the uncertainty of the transformation will increase with lesion size, especially if a nonlinear registration is used (Andersen et al., 2010; Ripolles et al., 2012; Jühling et al., 2024). This effect could be overcome either with cost-function (i.e., lesion) masking or enantiomorphic registration (Brett et al., 2001; Nachev et al., 2008). Both techniques provided good spatial normalization accuracy when dealing with singular lesions and would be easy to integrate with arT (**Figure 1C**) (Crinion et al., 2007). Even better results could be achieved with the use of additional study-specific templates, which were shown in patients with stroke or multiple sclerosis (Ashburner, 2007; Ripolles et al., 2012; Pirzada et al., 2020; Pappas et al., 2021). This way, even longitudinal studies of patients with brain tumors were possible (de Groot et al., 2023; Leenders et al., 2025). All of these techniques are provided, for example, with various SPM-toolboxes, like the LST Toolbox, especially suitable for lesions associated with diabetes, Alzheimer’s dementia, and multiple sclerosis, or the Clinical Toolbox, suitable for stroke lesions (Rorden et al., 2012; Schmidt et al., 2012; Schmidt, 2017). Since these techniques are compatible with our approach, arT appears even more promising, as the all-over acceptable good performance of generic ROI assessment could further increase the possibilities of more un-biased longitudinal investigations of focal brain lesions when assessed with quantitative perfusion parameters.

### 4.5 Limitations

We identified two major confounding sources of the proposed arT-method:

- Spatial resolution: A key limitation of this study was the rather low spatial resolution of the DSC technique in the z-direction, which directly seems to reduce the segmentation accuracy of the ROI segmentation. This was especially observed in ROIs with structures depicted only in a low number of slices (e.g., basal ganglia). Nevertheless, the errors encountered in the measurement of perfusion parameters using the generic ROIs with the proposed arT-method remained in an acceptable range. However, some improvement may also be achieved by a reduction in the slice thickness of the employed T2*-sequence. Since the slice thickness also has a direct impact on the contrast-to-noise ratio, decreasing the measured slice thickness should be done carefully and requires further investigation of the proposed method.
- Low tissue contrast: The native ROIs were segmented in the mean image of the realigned DSC sequences, which are usually of rather low contrast, rendering them rather prone to errors when manually segmenting structures like the basal ganglia or, e.g., infarct volumes. When using a co-registered anatomical space-defining sequence as an aid for segmentation, interpolation artifacts or partial volume effects must be considered. Here, using a high-resolution T1w high-contrast template with linear co-registration before performing arT could be advantageous. Anyway, this was not tested excessively in this study since all examinations were performed in a timely, optimized fashion, because of the need to rapidly detect a possibly critical ischemic perfusion state in our patients.

## 5 Conclusion and outlook

Analyzing quantitative perfusion-MRI parameters using generic ROIs in the brain is reliably feasible with the proposed arT-method. Concerning the relative bias and precision of the measurements, the results found for the arT-method were similar to other studies performed on the analysis of automatic segmentations. Moreover, our study showed lower relative variability in perfusion analysis than comparable studies. Therefore, arT could significantly speed up further scientific perfusion analyzes in large patient samples, while offering the possibility of analyzing functional data in native space, thereby avoiding any manipulation of the originally measured quantitative parameter values. However, the robustness of arT in pathological conditions still needs to be fully assessed, and further research on the accuracy of our approach in quantitative perfusion analysis of brain lesions is necessary. Nevertheless, arT could become a valuable tool, at least, for the assessment of multiple DSC-P-MRI measurements for routine and scientific purposes.

## Data Availability

The raw data supporting the conclusions of this article will be made available by the authors, without undue reservation.
